# Differential decay kinetics of human cytomegalovirus glycoprotein B genotypes following antiviral chemotherapy^☆^

**DOI:** 10.1016/j.jcv.2012.01.015

**Published:** 2012-05

**Authors:** Vincent C. Emery, Oriol Manuel, Anders Asberg, Xiaoli Pang, Deepali Kumar, Anders Hartmann, Jutta K. Preiksaitis, Mark D. Pescovitz, Halvor Rollag, Alan G. Jardine, Christoph G. Gahlemann, Atul Humar

**Affiliations:** a Centre for Virology, Department of Infection, University College Medical School, London, United Kingdom; b Transplant Infectious Diseases, University of Alberta, Edmonton, Canada; c Department of Pharmaceutical Biosciences, School of Pharmacy, University of Oslo, Oslo, Norway; d Department of Medicine, Rikshospitalet-Radiumhospitalet Medical Centre, University of Oslo, Oslo, Norway; e Department of Surgery, Indiana University, Indianapolis, IN, United States; f Institute of Microbiology, University of Oslo, Norway; g Department of Medicine, University of Glasgow, Glasgow, United Kingdom

**Keywords:** Viral replication, Ganciclovir, Fitness, Solid organ transplantation

## Abstract

**Background:**

The impact of different cytomegalovirus (HCMV) glycoprotein B (gB) genotypes on pathogenesis remains controversial.

**Objectives:**

To investigate the effect of gB genotypes either as single infections or as part of multiple infections on the early kinetics of response to ganciclovir therapy.

**Methods:**

Patients (*n* = 239) enrolled in a study of intravenous ganciclovir or valganciclovir for the treatment of HCMV disease were analysed by a gB genotype specific PCR to quantify the amount of each gB genotype present at initiation of therapy (baseline, day 0) and at days 3, 7, 14 and 21 post therapy.

**Results and conclusions:**

In all gB groups (individual gB genotype infections and mixed genotype infections) there was a biphasic decline in viral load after therapy. The first phase half life (days 0–3) was ≤1 day and was followed over the next 18 days by a slower second phase decline with half lives ranging from 3.4 to 4.4 days. The 1st phase rapid decline in viral load was dependent upon gB genotype whereas the ultimate viral load reduction at day 21 was relatively insensitive to gB genotype. A strong correlation between 1st phase decline and extent of viral load reduction at day 21 was observed (*r* = 0.37; *p* = 0.002). These data imply that early reductions in HCMV load after therapy may be useful in predicting the duration of drug therapy needed to control HCMV replication.

## 1 Introduction

Human cytomegalovirus (HCMV) remains an important infectious complication for the immunocompromised host. A range of direct and indirect effects have been associated with active replication (reviewed in 1, 2). Viral pathogenesis is directly related to the degree of viral replication with a number of studies showing viral load, and more recently cumulative load experienced during the period of replication, are diagnostic and prognostic markers of recurrent infection and disease.^3–5^ Complementary immunological studies indicate that the quality of CD4 and CD8 T-cell responses are critical factors in the control of high level replication.^6–12^ HCMV replication in vivo is highly dynamic with doubling times of approximately 1 day^13^ with a basic reproductive number in liver transplant recipients experiencing primary infection of approximately 15.^14^

At present, antiviral chemotherapeutic control of replication relies upon prophylactic deployment of valganciclovir (VGCV) and treatment of asymptomatic or symptomatic replication with either intravenous ganciclovir (iv GCV) or VGCV (reviewed in 15–17). There remains a paucity of data on the role of different HCMV strains in pathogenesis, and their response to immune or antiviral mediated control. Although the prototype laboratory adapted AD169 strain was originally sequenced in 1989^18^ and re-sequenced with the Towne strain more recently,^19^ only a limited number of clinical strains have been subjected to full genomic sequence analysis.^20,21^ However, various genes have been subjected to more intense sequence analysis at a macro and micro-scale including the surface glycoproteins B and H and UL139, UL144, UL147 and UL148.^22–28^ In the context of gB, four genotypes have been characterized based upon RFLP analysis.^29^ Although gB plays a critical role in HCMV entry and cell-to-cell spread,^30^ the clinical relevance of these gB genotypes remains controversial.^31–35^ At present, the majority of these analyses have taken place in relatively small numbers of patients infected with a single gB genotype. However, we now know that multi-genotype infections are relatively common^36,37^ and we reasoned that genotype specific declines in these mixed infections may provide new insight into the HCMV replication dynamics. The recently completed VICTOR study comparing iv GCV and VGCV for the therapy of HCMV syndrome and disease provided a large database of samples with frequent viral load sampling and a source for gB genotype analysis.^38^ Although we have previously reported on the epidemiology and clinical response rates with gB genotypes^39^ the present study undertakes an in-depth viral kinetics analysis to investigate the potential for differential decay kinetics of different gB genotypes either alone or when in competition with other gB genotypes and to ascertain whether early viral kinetics are associated with ultimate control of replication.

## 2 Materials and methods

### 2.1 Patient population and definitions

Solid organ transplant recipients enrolled in a randomized (1:1), open-label, parallel group, active drug-controlled multicentre and non-inferiority trial comparing treatment with oral valganciclovir to intravenous ganciclovir for the treatment of HCMV disease in solid organ transplant recipients (ClinicalTrials.gov NCT00431353) (VICTOR study) were included as previously described.^38^ A total of 321 patients received at least one dose of assigned medication with 164 patients randomized to treatment with 900 mg twice daily valganciclovir and 157 patients to 5 mg/kg twice daily i.v. ganciclovir included in the intention-to-treat population.^38^ Of these, 259 patients had confirmed HCMV viremia and made up the per-protocol population. It is this population in which gB genotype analysis was performed. It is important to note that patients in this study must have been diagnosed with HCMV disease prior to enrolment and that initiation of antiviral therapy for HCMV was not based on virologic markers. Both therapeutic drug formulations were administered for an induction period of 21 days, followed by 900 mg daily valganciclovir until day 49. Whole blood samples for viral load monitoring were obtained at the start of therapy (day 0, baseline) and at days 3, 7, 14 and 21 i.e. when patients are receiving full dose medication.

### 2.2 Glycoprotein B genotyping

Quantitative genotyping of glycoprotein B was performed by quantitative real-time PCR on DNA extracts from whole blood in all patients at days 0, 3, 7, 14, and 21 as described in detail elsewhere.^40^ A mixed infection was defined as HCMV infection with more than one gB genotype in a single sample.

### 2.3 Kinetics of viral load decline

Given that the results of the VICTOR study showed no differences between the treatment arms, we combined both groups for the analysis of the response of gB genotypes to therapy in either single gB genotype or in the context of mixed gB genotype infections. The kinetics of decline of HCMV load for each genotype within the mixed gB infection population was analysed separately using the mean log HCMV load at days 0, 3, 7, 14, 21. Decline rates were modelled using linear regression analysis and the decline rate constant computed using the formula:

(1) $$\text{Decline}\;\text{rate}=\frac{\ln\;\text{VL}(t_{1})-\ln\;\text{VL}(t_{2})}{t_{2}-t_{1}}$$

where VL is the HCMV gB genotype load at time *t*_1_ or *t*_2_ respectively.

Half lives of decline could then be computed using the following:

(2) $$T_{1/2}=\frac{\ln\;2}{\text{decline}\;\text{rate}}$$

Comparisons of the different rates of decline were performed using Student's *t*-test. The correlation between slope of decline and viral load reductions from baseline to day 21 was assessed using Spearman's rank correlation test. All *p*-values <0.05 were treated as significant.

## 3 Results

### 3.1 Patient characteristics and baseline HCMV load in patients with different gB genotypes

The gB genotype was determined for 239/259 of the per-protocol patients with HCMV disease enrolled in the VICTOR study where patients were randomized to receive either valganciclovir or intravenous ganciclovir at full dose to control their clinical symptoms of HCMV infection. At the initiation of antiviral therapy (day 0, baseline), the frequency each gB genotype was as follows: gB1 (61/239 (26%), gB2 (23/239 (10%), gB3 (24/239 (10%), gB4 (13/239 (6%) and mixed gB genotypes (118/239 (49%) [described in detail in 39]. Within the mixed infection population, the frequencies of the combinations were as follows: gB1/gB2 (*n* = 19), gB1/gB3 (*n* = 27), gB1/gB4 (*n* = 7), gB2/gB3 (*n* = 11), gB2/gB4 (*n* = 4), gB3/gB4 (*n* = 7), a mixture of three gB genotypes (*n* = 35) and all four genotypes (*n* = 8). There were no significant differences in age, gender or antiviral treatment received (intravenous GCV or VGCV), type of organ transplanted and HCMV serostatus when stratified according to gB genotype.

Baseline HCMV load i.e. at the initiation of treatment, in whole blood was highest in patients with mixed gB genotype infections (5.37 ± 0.92 log10 genomes/ml) compared to individual gB genotype infections although this was only significant when compared with gB1 and gB2 baseline HCMV loads (4.65 ± 0.93 log 10 genomes/ml (*p* = 0.0001) and 4.69 ± 0.85 log 10 genomes/ml (*p* = 0.002) respectively). In addition, baseline HCMV load for gB1 infections were significantly lower than both gB3 (5.32 ± 1.33 log10 genomes/ml; *p* = 0.008) and gB4 infections (5.25 ± 0.8 log10 genomes/ml; *p* = 0.04). Within the mixed gB genotype population, HCMV loads were comparable for gB2, gB3 and gB4 (4.65 ± 1.17 vs 4.64 ± 1.18 vs 4.59 ± 0.89 log10 genomes/ml respectively) but gB1 HCMV loads (4.27 ± 1.17 log10 genomes/ml) were significantly lower (*p* = 0.05).

### 3.2 Decay kinetics of gB genotype load in mixed and single infections after initiation of therapy

**Fig. 1:**
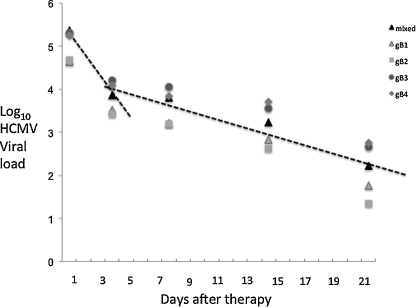
Decline in HCMV load in whole blood of patients with mixed gB genotype infections following receipt of ganciclovir therapy. The mean of each dataset at days 0, 3, 7, 14 and 21 were used to produce an average viral load at each time point (±1 SD) and a biphasic curve fit use to identify the mean rate of decline between days 0–3 and 3–21.

Initially we investigated the decay kinetics of HCMV in patients with only mixed gB genotype infection (*n* = 118) for both total HCMV load and for individual gB genotypes within the patients with mixed genotype infections. The decay kinetics for the total HCMV load followed a biphasic decline with an initial phase from days 0 to 3 having a half life of approximately 0.79 days and a slower 2nd phase decline between days 3 and 21 of 4.11 days (Fig. 1). Individual 1st and 2nd phase kinetics for each gB genotype differed within this mixed gB genotype population (Table 1). gB genotype 1 was associated with the slowest 1st phase decline (1.04 days) which was significant for the comparison between gB1 and gB2/gB4 (*p* = 0.03 and 0.015 respectively). In addition, both gB2 and gB4 1st phase half lives were significantly more rapid than the gB3 1st phase half life (*p* = 0.01 for both comparisons). In contrast to these differences, the 2nd phase decline rates were more comparable with half lives of approximately 3.5 days with only a significant difference observed between the 2nd phase decline of gB2 and gB3 infections (*p* = 0.04).

In order to place these individual gB genotype decay kinetics within the mixed gB genotype population in context, we assessed the HCMV decay kinetics of patients with single gB genotype infections (*n* = 121). Consistent with our observations for the mixed gB genotype decay kinetics, single gB genotype infections followed a biphasic decline with a 1st phase half life of approximately 1 day and a second phase decline rate of between 3.5 and 5 days (Table 2). Comparison of the 1st and 2nd phase half lives between patients with single gB genotype infections and mixed infection revealed that the 1st phase half life for all mixed infections were significantly faster compared to gB1 (difference = 0.38 days (95% CI 0.08–0.67); *p* = 0.013), gB3 (difference 0.38 days (95% CI 0.04–0.71); *p* = 0.028) and gB4 (difference = 0.4 days (95% CI 0.05–0.75); *p* = 0.025). In contrast, there were no significant differences in the 2nd phase decline between single gB genotype infections and the mixed infection group.

### 3.3 Correlation between decay kinetics and replicative control at day 21

**Fig. 2:**
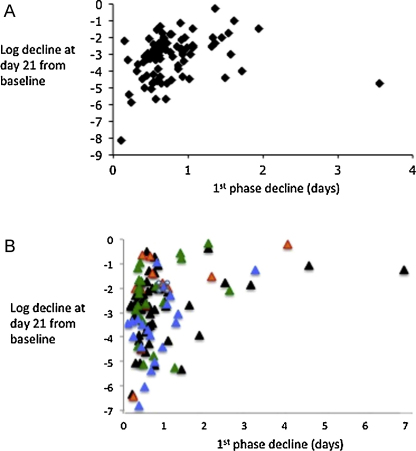
Relationship between the first phase decline half life and log reduction in HCMV load attained at day 21 of therapy. The data are shown for patients with mixed gB genotype infection (panel A, *n* = 118) and for patients with single gB genotype infections (panel B). Individual gB genotypes in panel B are identified by the colour of the marker (gB1 (*n* = 42) = black; gB2 (*n* = 20) = blue; gB3 (*n* = 23) = green and gB4 (*n* = 12) = red). The Spearman's rank correlation coefficient of the data in panel A was 0.37 (*p* = 0.002) and for the data in panel B was 0.35 (*p* = 0.0004). (For interpretation of the references to colour in this figure legend, the reader is referred to the web version of the article.)

We next investigated whether the 1st phase decline kinetics of HCMV in whole blood after initiation of therapy was associated with either the 2nd phase decline rate or the absolute reduction in HCMV load by day 21 of therapy. There was no correlation between 1st and 2nd phase decline rates in any groups (mixed gB genotypes or single gB genotype patients). In contrast, there was a significant correlation between 1st phase decline rates and the log reduction in HCMV load between day 0 and day 21 in patients with mixed gB genotype infection (Spearman's *r* = 0.37; *p* = 0.002) or when the single gB genotype infections were combined (Spearman's *r* = 0.35; *p* = 0.0004; Fig. 2). When the individual gB genotypes were analysed separately the 1st phase decline in gB1, gB2 and gB4 infections was significantly correlated with the log decline at day 21 (*r* = 0.32 (*p* = 0.02); *r* = 0.45 (*p* = 0.04); *r* = 0.67 (*p* = 0.02) respectively) whereas the same analysis for gB3 failed to reach statistical significance (*r* = 0.22; *p* = 0.28).

## 4 Discussion

To date there have been relatively few large scale analyses of the in vivo effect of antiviral therapy on different HCMV strains. In the present study we show that subtle differences in the early kinetics of response to antiviral chemotherapy are apparent between different gB genotypes and in patients with mixed gB genotype infections. However, after 21 days of therapy these differences were insignificant i.e. gB genotype appears not to influence the ultimate control of replication after ganciclovir therapy. An important observation in our study was that HCMV load in whole blood appears to follow a bi-phasic decline with an initial half life of <1 day and a second phase half life of about 4 days. This biphasic decline has been recently been described in a single case report of a stem cell transplant recipient after artesunate therapy.^41^ Previous work in HIV-infected HCMV retinitis patients where HCMV load was in a quasi-steady state has shown that HCMV replication is highly dynamic with half life of decline averaging 1 day.^13,14^ Thus, the first phase decline observed in our present study would be consistent with this data.

Exploration of the factors associated with the different decline rates and ultimate control of replication i.e. baseline viral load in individual or mixed gB genotype infections, the relationship between 1st and 2nd phase declines etc. did not reveal any significant associations. However, there was a significant association between the half life of the 1st phase decline and the ultimate log reduction in HCMV load attained at day 21 of therapy in patient with mixed gB genotype infections and when all single gB genotype infected patients were combined. To our knowledge this is the first report of an association between very early viral load kinetics after anti-HCMV therapy and the ultimate antiviral control of HCMV replication at later times. These observations are consistent with data for both HIV and HCV treatments indicating that the very early viral kinetics provide important prognostic information for ultimate replication control and sustained virological response.^42–44^ In addition, it has been reported previously that higher viral loads at baseline are a risk factor for failure to control CMV replication below detectable levels in patients included in the present study and that patients with mixed gB genotype infections have higher baseline viral loads.^39^

It is unclear why polymorphisms in the gB gene might be associated with differences in antiviral response. Although gB plays important roles in viral binding and entry to cells^30^ it is possible that it may be a surrogate marker for other genetic traits i.e. gB genotypes are in linkage disequilibrium with distinct DNA polymerase genotypes that are associated with more or less replication competent viruses especially since the polymerase is adjacent to gB on the HCMV genome. This could account for the differences seen in our study within individual gB genotype populations. Although superinfection with a new strain would be facilitated by the immune evasion genes of the incoming HCMV strain^45^ we do not think that this has a major influence on response to therapy and in other studies, serostatus has not been associated with different decline rates.^46^ However, the interaction between gB genotypes during a mixed infection including competition and relative fitness differences may also contribute to our observations. In order to disentangle this area, whole genome sequencing, or deep sequencing^47^ of the HCMV strains present in these patients will be informative and should further enhance our knowledge of the genetic fluidity of pathogenic strains of HCMV and allow more sophisticated dynamic models of HCMV replication to be developed.

## Conflicts of interest

The following authors have received honoraria from Roche Pharmaceuticals for advisory boards and presentations: Vincent C. Emery, Anders Asberg, Deepali Kumar, Anders Hartmann, Mark D. Pescovitz, Halvor Rollag, Alan G. Jardine and Atul Humar.

## Figures and Tables

**Table 1:** Biphasic decline parameters for each gB genotype in patients with mixed gB genotype infections.

Genotype (*n*)	Baseline HCMV load (log_10_ genomes/ml)	1st phase half life of decline (days)	2nd phase half life of decline (days)
gB1 (*n* = 61)	4.65 ± 0.95	1.04 ± 0.81	3.66 ± 2.28
gB2 (*n* = 23)	4.69 ± 0.85	0.65 ± 0.27	4.36 ± 3.31
gB3 (*n* = 24)	5.32 ± 1.33	0.94 ± 1.0	3.46 ± 2.39
gB4 (*n* = 13)	5.25 ± 0.92	0.65 ± 0.35	3.40 ± 1.86

**Table 2:** Biphasic decline parameters for single gB genotype infections and cumulatively for patients with mixed gB genotype infections. These decline rates for gB 1–4 should be compared with the gB genotype declines rates in the patients with mixed infections shown in Table 1.

Genotype	Baseline HCNV load (log_10_ genomes/ml)	1st phase half live of decline (days) (number, *n*)	2nd phase half life of decline (days) (number, *n*)
gB1	4.27 ± 1.17	1.17 ± 1.31 (*n* = 56)	4.27 ± 2.77 (*n* = 47)
gB2	4.65 ± 1.15	0.92 ± 0.78 (*n* = 20)	3.62 ± 2.83 (*n* = 19)
gB3	4.64 ± 1.18	1.17 ± 1.36 (*n* = 23)	5.25 ± 3.96 (*n* = 22)
gB4	4.59 ± 0.89	1.19 ± 1.09 (*n* = 12)	5.08 ± 3.34 (*n* = 10)
Mixed infection	5.37 ± 0.92	0.79 ± 0.67 (*n* = 87)	3.84 ± 3.28 (*n* = 81)
